# Is this a man’s world? The effect of gender diversity and gender equality on firm innovativeness

**DOI:** 10.1371/journal.pone.0222443

**Published:** 2019-09-18

**Authors:** Daniela Ritter-Hayashi, Patrick Vermeulen, Joris Knoben

**Affiliations:** 1 Department of Business Administration, Radboud University Nijmegen, Gelderland, The Netherlands; 2 Department of Economics and Business Economics, Radboud University Nijmegen, Gelderland, The Netherlands; The Bucharest University of Economic Studies, ROMANIA

## Abstract

Gender diversity is known to have a positive effect on innovation in developed countries. However, it is unclear whether the benefits of gender diversity for innovation also apply to the particular context of developing countries, which is characterized by diverse and lower levels of gender equality. We propose that gender diversity positively impacts innovation in the developing countries participating in our study. In addition, we expect that this effect is moderated by country-specific levels of gender equality. In a cross-country study covering 18,547 firms in 15 developing countries, we find that gender diversity among a firm’s owners and workforce as well as having a female top manager benefit innovation in developing countries. Yet, contradictory to our expectations, gender equality does not significantly moderate this relationship. As such, our results underline the importance of enabling and fostering gender diversity and have critical implications for firms and policy makers alike.

## Introduction

Gender diversity, which refers to the balance between the two genders [1], is frequently found to have a positive effect on innovation in developed countries [1–4]. More specifically, gender diversity facilitates innovation due to a more diversified knowledge pool [5–7] and improved decision making [1,8]. Despite the importance of innovation for economic growth and poverty reduction in developing countries [9] and an increasing understanding of innovation in this specific context [10,11], gender diversity as a means to foster innovation in developing countries has thus far received no attention in the literature.

We expand existing research from developed to developing countries and expect that gender equality strongly influences the extent to which firms can reap the proposed benefits of gender diversity for innovation. Gender equality is societally engrained, manifested in country policy and laws as well as visible in practices such as access to education [12]. We propose that with increasing levels of gender equality, firms can benefit more from the advantages of gender diversity for innovation. On the one hand, and in relation to the first abovementioned benefit of gender diversity (knowledge diversification), gender equality entails whether policy and practice grant boys and girls as well as men and women equal access to education [13]. Education is considered to be an important driver for knowledge [14] and we expect the benefit of knowledge diversification for innovation [6,15] to be limited if women are deprived from education. On the other hand, the societally engrained perception and status women are associated with is an important difference between countries. Gender is one of the observable attributes related to status beliefs that are powerful in organizing the patterns of respect and influence among individuals as they interact in decision making [16]. In a societal context where women are attributed with a low status, their contribution to the decision making process (second above referenced benefit of gender diversity) is expected to be disregarded [17].

Gender equality levels in the developing countries in Africa, the Middle East and South Asia participating in this study are much lower than in the developed countries where the positive effect of gender diversity on innovation has been previously established [9]. Given the importance of innovation in developing countries (5,6), our focus on gender diversity is relevant for both firms as well as for policy makers.

Our contributions are threefold. First, gender diversity has not been assessed as a potential firm-level mechanism to increase innovation in developing countries. Previous innovation-related studies in developing countries have focused on e.g. the types of innovation [10], the role various knowledge sources play in explaining innovation [11], as well as firm-level differences steering incremental versus radial innovation [18]. We extend knowledge on the relationship between gender diversity and innovation from developed to developing countries. Moreover, we distinguish between gender diversity at different organizational levels: gender diversity of firm owners and workforce and the gender of the highest-ranking manager. As such, our research answers the frequent request to empirically assess developing countries ‘[…] as a research context, by demonstrating how […] understudied phenomena influence existing theories as well as how newly emerging phenomena have the potential to generate new theories in management research’ [19].

Second, gender equality has thus far not been taken into consideration as a potential contingency factor when determining the effect of gender diversity on innovation. Our results suggest that innovation benefits from gender diversity *independent* of country-level gender equality, which further signals the importance of gender diversity for the innovative potential of firms in developing countries.

Third, our study has high managerial relevance at a firm-level and is essential for innovation-targeted policy making on a country level. The degree of a firm’s gender balance is within the merits of managerial decision making and management driven strategic initiatives [20]. By contributing to an in-depth understanding of whether and how gender diversity can benefit innovation in the specific context of developing countries, our study enables informed managerial decision-making aimed at increasing innovation. Moreover, given the importance of finding ways to foster innovation in developing countries as a driver for economic growth and poverty reduction [9], our research provides interesting insights for policy makers. It points them to the importance of ratifying and enforcing policies targeted at increasing gender diversity at all hierarchical levels in the firm as a means to foster innovation.

The remainder of this article is structured as follows. We first provide an overview of the theoretical background and develop our hypotheses. Next, the empirical data and research methodology are presented. Subsequently, we describe the analysis conducted, followed by a summary of the results. Finally, we discuss our findings and provide closing remarks and conclusions.

## Gender diversity and innovation

Innovation refers to the ‘generation, acceptance and implementation of new ideas, processes, products or services’ [21]. It is an important driver for increasing firm performance, enhancing competitive advantage and expanding market share [4] as well as for the economic growth of countries [22]. In this study we focus on one of the five types of innovation classified by Schumpeter, namely product innovation. Product innovation is defined as the introduction of new goods or services as well as the significant improvement of existing products with regards to their characteristics and intended use [1]. Innovation in developed countries has frequently been found to benefit from gender diversity [1–4,23], which, according to the relational demography theory, is regarded upon as a compositional characteristic of groups [24]. As part of the value in diversity perspective [25], we discuss important benefits of gender diversity for innovation, stemming ‘either from each member’s unique attributes which bring different perspectives to the group or from relational and motivational processes that occur in diverse groups’ [23].

First, gender diversity expands the knowledge, which is available to a firm both internally as well as externally: Given their different experiences and career trajectories, the human and social capital of men and women [5], including their cognitive schemes and as well as systems of meaning, differ. Even though an initial investment of diverse employees to create a shared language and understanding is required, they consequently benefit from information and resources they would otherwise not have access to [26]. As such, gender diversity enriches the internally available knowledge stock. Moreover, based on the social network theory [27] and the self-categorization theory [28], people typically direct their networking strategies to individuals with similar demographics [29]. Consequently, gender diversity provides firms with access to different external networks and with that to a diversified knowledge pool outside the firm [7]. As diverse knowledge can be complementary, enables new combinations of knowledge and ultimately benefits the creation of new ideas and products, this diversified internal and external knowledge stock increases the innovation levels within a firm [6].

Second, gender diversity can benefit innovation as it is linked to improved problem solving and decision making [1]. Successful innovation requires well-founded decisions on which innovative ideas to drop and which to turn into tangible innovative outputs [8]. Cognitive conflict and expression of diverse viewpoints stemming from men’s and women’s inherently different perceptions and experiences [5] can circumvent premature consensus and thus increase the quality of decisions [30] as a driver for innovation.

We argue that the extent to which firms can take advantage of the above elaborated on benefits of gender diversity for innovation depends on the country-specific levels of gender equality. Gender equality refers to girls and women having ‘equal access to quality education, economic resources and political participation as well as equal opportunities with men and boys for employment, leadership and decision-making at all levels’ [31]. Gender equality spans across social, political, and economic dimensions in that it is manifested in country policy, deep-seated in society and culture [12] as well as visible in e.g. girls’ and women’s access to education and their social status [13]. We propose that operating in a country with higher levels of gender equality enhances a firm’s ability to reap the benefits of gender diversity among the firm owners and the workforce for innovation compared to operating in countries with little gender equality. Moreover, we expect gender equality to impact the relationship between having a female top manager and innovation. We elaborate on the three proposed interaction effects in the following sections.

### Gender equality and gender diversity in the ownership structure

A firm’s owners, which can refer to single individuals, a group of individuals or a board of directors representing shareholders [32], directly influence innovation through the strategic decisions they take [33]. In the decision making process among owners, gender-related diversity plays an important role as outlined by Hambrick and Mason’s [34] upper echelon theory. Firm leaders filter and interpret the stimuli they receive during a decision challenge based on their personal and unobservable cognitive bases and values, for which demographic attributes such as gender build the foundation [35]. Despite a general preference for homogeneity [36], gender diverse owners ‘can make a valuable contribution to […] decisions by providing unique perspectives on strategic issues’ [37]. Hence, by fostering cognitive dissent [8] and avoiding premature consensus [30], diversity in general [38] and gender diversity in specific are frequently found to benefit innovation [2,4] through improved problem solving and enhanced decision making [1].

However, the extent to which firms can reap the aforementioned benefits of gender diverse owners on innovation may depend on gender equality. Gender equality subsumes the societally established legal and social status of women, which determines the patterns of respect and influence among individuals as they interact and take decisions [16]. When women have low social status in a gender unequal context, ‘the potential contributions of women stemming from their different values are likely to be disregarded’ [17] in the decision making process. However, with increasing levels of gender equality, we expect the opinions, knowledge and experiences of both men and women to be equally taken into consideration. Thus, we infer that the positive effect of gender diversity among a firm’s ownership structure on innovation will be more pronounced in countries with high levels of gender equality compared to countries with low levels of gender equality. We hypothesize the following:

***Hypothesis 1a***. *Gender diversity in a firm’s ownership structure has a positive effect on its likelihood to innovate*.
***Hypothesis 1b***.*The effect of gender diversity in a firm’s ownership structure on its likelihood to innovate is positively moderated by gender equality in a country*.

### Gender equality and the gender of the top manager

Top managers influence firms’ innovativeness [39] as they are instrumental in building an innovation-nurturing organizational climate, which includes mutual trust as well as employee involvement and accountability [40]. Particularly transformational leaders ‘inspire, energize, and intellectually stimulate their employees’ [41]. They rely on motivational processes which are both longer-term as well as vision-based [42] and thereby ‘stir their employees to look beyond their own self-interest for the good of the group’ [41]. Transformational leadership is conducive to innovation [40,42–44] and has previously been established to be more frequently exhibited by women than by men [45]. One could infer that especially women top managers have the potential to advance firms’ innovation levels. Yet, studies assessing the relationship between a top manager’s gender and innovation are scarce and have primarily been conducted in developed countries. Studies that have included the top manager’s gender, mostly focused on CEOs (Chief Executive Officers) as the highest ranking management individual in large organizations [46]. The limited research available yields in inconsistent [46] or insignificant [47] results. As such, it is important to enhance our understanding of this relationship in the context of developing countries.

Beyond a possible direct effect, we expect that gender equality affects the extent to which female top managers can drive innovation. A study by Wolfram and colleagues [48] gives a first indication that the gender of managers and the therewith associated status can play an important role in the interaction with their followers. Their findings suggest that male followers respect female leaders less than male leaders if they believe in traditional gender roles. This lack of respect can limit a firm’s innovativeness, which according to the leader member exchange (LMX) theory [49], depends on a good relationship and quality exchange between leaders and their followers [50]. Along the same lines, Lyngsie and Foss suggest that ‘employees’ stereotyping and prejudice may negatively influence positive effects of increasing female participation in […top management teams]’ [51]. Hence, we claim that gender equality moderates the relationship between a top manager’s gender and a firm’s likelihood to innovate. We formulate the following hypotheses:

***Hypothesis 2a***. *The gender of the top manager has a direct effect on a firm’s likelihood to innovate*.
***Hypothesis 2b***. *The effect of the top manager’s gender on a firm’s likelihood to innovate is positively moderated by gender equality in a country*.

### Gender equality and gender diversity in the workforce

Innovation can also be initiated and driven by the employees of a firm. In bottom-up innovation processes, employees draw upon their individual pool of knowledge and experience to contribute to different activities in the innovation process [52]. Both knowledge at the level of individuals and shared knowledge at group and divisional levels are critical for innovation [53]. As explained earlier, gender diversity has been found to benefit innovation in developed countries [1,54] by diversifying the internal [6] and external knowledge pool [7]. For example, a study by Díaz-García et al. demonstrates that gender diversity within R&D teams results in high levels of radical innovation, especially in technology intensive industries [23]. Moreover, Teruel and colleagues find that ‘gender-diverse teams increase the probability of innovating, and this capacity is positively related to team size’ [3]. We also expect that this positive effect of gender diversity among the workforce on innovation can be found in developing countries. The benefits of a diverse pool of knowledge in the workforce allows for unique and novel combinations of knowledge that support firms in developing countries to unleash their innovative potential (9).

Furthermore, we expect a higher degree of gender equality, and with that enhanced access for girls and women to education [13] and knowledge, to enable a better realization of the benefits gender diversity can bring for innovation. The more women and men can contribute equal levels of inherently different knowledge and experiences to a firm’s knowledge pool, the more diverse the knowledge pool becomes, which in turn is a driver for firm innovation. Hence, we infer that the positive effect of gender diversity on innovation will be more pronounced in countries with high gender equality compared to countries with low equality between men and women. We formulate the following hypothesis:

***Hypothesis 3a***. *Gender diversity in a firm’s workforce has a positive effect on its likelihood to innovate*.
***Hypothesis 3b***. *The effect of gender diversity in a firm’s workforce on its likelihood to innovate is positively moderated by gender equality in a country*.

## Data and method

### Data

We test our hypotheses using a combination of the World Bank’s Enterprise Survey (ES) and the Women’s Economic Opportunity Index (WEOI) of the Economist Intelligence Unit [13]. The ES is a firm-level survey, encompassing firms in the manufacturing, retail and service industry. It covers three main components, namely firm characteristics, the business environment as well as innovation related activities. It is stratified based on firm size, geographical location and industry sector. The ES questionnaire is answered by top managers or business owners, with the support of company accountants and human resource managers if required for specific questions. We focus on developing countries in Africa, the Middle East and South Asia. The selection of countries for this study is based on three criteria, namely: the status of being a developing country (low or lower-middle income as per World Bank definition in 2012), the period of ES data collection (2013 and 2014), and the availability of all required variables, including the measurement of gender equality (Women’s Economic Opportunity Index, WOEI). As a result, 15 countries (Bangladesh, Egypt, Ghana, India, Kenya, Malawi, Morocco, Nigeria, Pakistan, Senegal, Sudan, Tanzania, Uganda, Yemen and Zambia) are included in this research. This combined data set, including in-depth insights on a high number of firms covered by the ES and a wide range of countries with information on gender equality, enables a country-based comparison of 15 countries with 18,547 firms.

#### Dependent variable

Our measure of innovation is based on the following ES questions: ‘During the last three years, has this establishment introduced new or significantly improved products or services?’. Affirmative answers are coded one, negative replies zero. Innovation in previous gender and innovation research has been both measured by objective measures such as the number of patents [32] as well as by measures very similar to the more subjective binary response of firm-level informants used in this study [3,4]. Despite its relative subjectivity, this measure of innovation is particularly suitable in the developing country context of this study, where innovation is frequently incremental rather than radical in nature and as such rarely results in the formal registration of patents [9]. The binary nature of our dependent variable itself bears inherent limitations, which are referenced in more detail in the discussion section.

#### Independent variables

**Blau’s Index**: Consistent with previous operationalization [2,3,46], we use Blau’s (1977) index of heterogeneity to assess gender diversity among a firm’s workforce and owners. Blau’s index, originally proposed by Simpson in 1949, is also known as the Herfindahl’s and the Hirschman’s index and it is considered to be an appropriate measure for capturing variations within a group of people [24]. Blau’s index is computed (1-∑p_k_^2^), where p is the proportion of group members in each of the k categories, and it ranges from 0.00 (only one gender) to 0.50 (equal numbers of men and women).

**Gender Diversity in the Workforce**: More specifically, the underlying data for computing Blau’s Index as a measure for gender diversity among the workforce, is based on the overall number of ‘permanent, full-time individuals’ and on the number of ‘permanent full-time individuals that [..] were female’ (ES).

**Gender Diversity among the Owners**: The measure of gender diversity among the owners of a firm specifically accounts for the emerging market context of this study, which is characterized primarily by small to medium sized firms without a formal board of directors. Our measure therefore differs from previous studies conducted in developed countries, which capture the percentage of women on the corporate board based on firms’ widespread preference for diversified shareholder models [2,4]. Our measure (Blau’s index) is based on the following two questions: ‘Among the owners of the firm, are there any females?’ and ‘What percentage of the firm is owned by females?’.

**Gender of the Top Manager**: The third independent variable captures the gender of a firm’s top manager, not gender diversity per se. The term top manager in this study refers to the firm’s highest-ranking management individual and it is measured by the following question: ‘Is the Top Manager female’. Positive responses are coded one, negative replies are coded zero.

#### Moderator: Gender equality

According to van Staveren [55], five gender indices are most widely used to measure gender equality given their reputable sources and high coverage. One of those five indices is the Women’s Economic Opportunity Index (WEOI) of the Economist Intelligence Unit [13], which we rely on in this study. The WEOI provides insights to which degree the ‘set of laws, regulations, practices, customs and attitudes […] allow women to participate in the workforce under conditions roughly equal to those of men’ (Economist Intelligence Unit, 2012, p.5).

The WEOI is used in this research as a measure for gender equality for several reasons. First, it covers gender equality very comprehensively as it encompasses all four dimension of human development (resources, institutions, capabilities and functionings) [55]. More specifically, the WEOI incorporates insights which are particularly relevant in the context of this study, such as policies and laws discriminating against women, their social and legal status as well as their access to education [13]. Second, it is a measure, which has been successfully used in previous studies on gender equality [56,57]. Lastly, the WEOI’s focus on resources and institutions is well suited for creating a better understanding of how business and economic law can be particularly restricting or discriminating against women [58] when ‘social customs and institutions effectively limit or prevent women’s access to most economic and political opportunities’ [59].

The WEOI incorporates several national and international sources, both quantitative and qualitative in nature. It consists of 29 indicators in five categories, which are summarized in tabular form in S1 Appendix. Category one, labor policy and practice, captures the degree to which governments ratify and enforce policies aimed at providing women with equal opportunities and pay. Category two, access to finance, focuses on women’s access to finance related programs and services as well as their ability to build credit history. Category three captures the education and training level of women. In category four, the WEOI entails information on women’s legal and social status. This includes violence against women, their citizenship and ownership rights, discrimination, political participation and fertility. The fifth category captures the regulatory quality of a country, touching upon the ease of starting a business, adequate infrastructure to do so as well as access to technology. The overall WEOI score, which ranges from 0–100 with higher values representing higher levels of gender equality, is computed from a simple average of the unweighted mean of each category’s indicators [13]. Table 1 encompasses an overview of the relevant WEOI values at the country level as well as a country’s rank in a world-wide comparison. The information required to calculate the internal reliability of the WEOI is not available. Consequently, the internal reliability of the WEOI cannot be reported.

**Table 1:** Gender equality measured by WEOI ratings and ranking for countries participating in this study.

Country ^a^	Region	WEOI Value ^b^	Rank ^c^
Egypt	Middle East & North Africa	48.7	80
Kenya	Sub-Saharan Africa	47.5	86
Morocco	Middle East & North Africa	47.0	89
Ghana	Sub-Saharan Africa	46.4	91
Tanzania	Sub-Saharan Africa	45.4	95
India	South Asia	41.9	98
Uganda	Sub-Saharan Africa	40.4	102
Bangladesh	South Asia	39.2	105
Malawi	Sub-Saharan Africa	39.0	107
Senegal	Sub-Saharan Africa	38.7	108
Zambia	Sub-Saharan Africa	36.9	112
Pakistan	South Asia	35.5	116
Nigeria	Sub-Saharan Africa	33.4	119
Yemen	Middle East & North Africa	24.6	126
Sudan ^d^	Sub-Saharan Africa	19.2	128

^a^ Classification World Bank for 2012: lower-middle income countries (GNI per capita of maximum US$4,085), except for Malawi Tanzania, Uganda (low income countries, GNI per capita of US$1,035 or less)

^b^ Maximum WEOI value: 100.0

^c^ Out of 128

^d^ Lowest WEOI score and rank worldwide

#### Control variables

**Firm size**: Based on prior findings indicating a positive relationship between the size of a firm and its innovation levels [1,23], we control for firm size by its number of full-time permanent employees (continuous variable, log-transformed).

**Firm Type**: Furthermore, we control for whether an ‘Establishment is part of a larger firm’ (ES) and with that in a position to benefit from economies of scale of the parent organization. If answered affirmatively, we coded establishments as one, otherwise zero.

**Export**: Export allows firms to ‘benefit from the access they gain to knowledge that resides abroad’[60]. In line with previous research establishing a positive effect of export on innovation [3,61], we moreover control for whether a firm generates sales from export. We coded a firm zero for national sales only and one for indirect and direct export.

**R&D**: Given R&D’s positive effect on innovation [1,3,62], we also control for whether firms ‘spend on formal R&D activities, either in-house or contracted with other companies’ during the last three years (ES). We coded a positive response one and a negative response zero.

**Education**: Preceding studies suggest a highly educated workforce to positively impact innovation [1,3]. Thus, we additionally control for employees’ education level by assessing the percentage of employees who completed secondary school (ES).

**Industry Sector**: In line with previous research [1,61], we account for the industry sector, in which a firm operates. Manufacturing was dummy-coded one, retail two and the services (non-retail) industry three.

**Ownership form**: Participating firms are characterized by different ownership forms, such as shareholding companies with privately traded or no shares (dummy-coded two), firms governed by sole proprietorship (three), partnerships (four), limited partnerships (five), and all other ownership forms (six). As insights into the gender of the owners of publicly traded shares are difficult to gather and thus not sufficiently reliable, firms with shares traded publicly were excluded from this research. As sole proprietorship does not allow for gender diversity among a firm’s owners, we have analysed the effect of a gender diverse ownership structure on innovation while both including and excluding firms governed by sole proprietorship. The coefficients and significance of the results remained constant to the variation in this variable specification.

To account for country specific characteristics, we additionally include the following control variables provided by the World Bank:

**Income Classification**: We control for the World Bank classification (2012) of countries as either being low income countries (GNI per capita US$ 4,085 or less) or lower-middle income countries (GNI per capita of maximum US$4,085). Firms classified as low-income countries are coded zero, lower-middle income countries are coded one.

**Employment in Agriculture**: We furthermore account for the percentage of a country’s population, which is employed in the agricultural sector (continuous variable). A country’s agricultural economic performance is critical for the reduction of extreme poverty on the one side, whilst a decreasing share of the agricultural sector in employment is a consequence of economic progress on the other side [63].

**Foreign Direct Investment (FDI) net inflow**: We additionally shed light on the percentage of FDI net inflow as a percentage of the overall GDP. FDI net inflow refers to the value of direct inward investment issued by investors residing outside of a particular country. It can be understood as a proxy for influence and control, which is excerpted from outside the country [64].

**Rural Population**: The term rural population refers to people living in rural areas [65]. The national standard for classifying areas as rural varies between countries and is determined based on population density and distance to large cities. We capture the percentage of the rural population among the total population for two primary reasons. First, the percentage of the population, which is living in rural areas, is frequently associated with higher levels of poverty [63]. Second and especially relevant for the dependent variable (product innovation), businesses operating in rural areas face pronounced operating challenges and are often prone to lower innovation levels compared to firms located in urban surroundings [66].

### Statistical analysis

Given the potentially clustered nature of our data at the country-level, the commonly known regression assumption of independence can be violated [67]. Different model specifications allow to account for the risk of heteroscedasticity. To analyze our data, we rely on a binary logistic regression model with clustered standard errors [67]. We further conduct a robustness test with multi-level modeling (hierarchical linear model) [68]. If not stated otherwise, the results of both model specifications are consistent and only the results of the main analysis (binary logistic regression model with clustered standard errors) are reported.

To account for the moderating effect of gender equality, we included interaction effects in the analysis, which potentially bears the risk of high correlation with lower order terms [69]. When testing for the previously described challenge we found that, without centering the independent variables and the moderator at their mean, tolerance was at times below .2 and VIF values above 10, with an average VIF value way beyond 1 ($\bar{VIF}=22.09$), indicating issues with multicollinearity [70]. After centering the previously mentioned variables at their respective mean, multicollinearity is of no concern: for all variables, Tolerance is above .2 and VIF values are smaller than 10 with the average VIF value not being substantially greater than 1 ($\bar{VIF}=1.32$). Thus, the decision to mean-center the independent variables and the moderator was taken.

## Results

The descriptive statistics and bivariate correlations for all variables are outlined in Table 2. The subsequent insights illustrate the general trends how and in which context the firms participating in our research operate. First, the majority are small to medium sized (75.34 percent) independent firms (79.79 percent) in the manufacturing industry (66.43 percent). Most firms, whose average percentage of employees having completed secondary school accounts for 50.12 percent, are not conducting R&D (75.66 percent) and generate their sales primarily from national transactions (81.99 percent). Second, gender diversity at all levels is rather low, expressed in a Blau’s index of 0.05 among firm owners and of 0.16 among the overall workforce. In other words and more illustrative, as much as roughly 91.49 percent of the firms’ owners and 84.82 percent of the firms’ workforce are men. These findings are in line with previous insights that women are under-represented both in the workforce as well as particularly in management or ownership positions in developing countries [58]. This also holds true for female representation among top managers: only 8.70 percent are women. Furthermore, the mean gender equality level derived for our study (WEOI, 40.50) is well below the worldwide average of 57.30. Nevertheless, there is still a considerable range in the gender equality values with a minimum of 19.23 for Sudan, representing the lowest WEOI worldwide, and a maximum of 48.70 for Egypt. The aforementioned results underline our initial observation that the level of gender equality in the countries participating in this study differs, however at generally lower levels compared to developed countries. Third, the dependent variable, innovation, indicates that 43 percent of the firms participating in this study introduced a new or significantly improved product or service.

**Table 2:** Descriptive statistics and correlation matrix (n = 18 547).

	Variable	Mean	Std. Dev.	Min	Max	1	2	3	4	5	6	7	8	9	10	11	12	13	14	15	16	17	18	19
1	Innovation	0.43	0.50	0.00	1.00	-																		
2	Industry (Manufacturing)	0.66	0.47	0.00	1.00	-0.01	-																	
3	Industry (Retail)	0.13	0.33	0.00	1.00	0.03	-0.54	-																
4	Firm Size (log-transformed)	89.13	355.59	1,00	21001	0.05	0.06	-0.03	-															
5	Firm Type	0.20	0.40	0.00	1.00	0.06	-0.04	0.03	0.15	-														
6	Export	0.18	0.38	0.00	1.00	0.11	0.10	-0.10	0.18	0.11	-													
7	R&D	0.24	0.43	0.00	1.00	0.35	0.07	-0.03	0.08	0.12	0.16	-												
8	Education	50.12	33.69	0.00	100.0	0.13	-0.18	0.13	0.04	0.05	0.00	0.13	-											
9	Ownership (Shareholding)	0.10	0.30	0.00	1.00	0.01	0.04	-0.06	0.13	0.10	0.16	0.06	0.01	-										
10	Ownership (Sole Proprietorship)	0.55	0.50	0.00	100.0	-0.06	-0.08	0.10	-0.13	-0.18	-0.19	-0.16	-0.09	-0.37	-									
11	Ownership (Partnership)	0.17	0.38	0.00	1.00	-0.03	0.07	-0.05	-0.01	0.03	0.01	0.01	-0.04	-0.15	-0.51	-								
12	Ownership (Limited Partnership)	0.16	0.36	0.00	1.00	0.10	0.00	-0.02	0.08	0.12	0.10	0.16	0.15	-0.14	-0.47	-0.20	-							
13	World Bank Classification	0.86	0.34	0.00	1.00	-0.03	0.03	-0.04	0.02	0.02	0.01	0.06	0.03	0.04	-0.10	0.05	0.05	-						
14	Employment in Agriculture	44.86	10.59	27.07	85.14	0.13	-0.02	0.04	-0.01	-0.03	-0.04	0.11	0.06	-0.06	0.01	-0.03	0.05	-0.41	-					
15	Foreign Direct Investment	1.88	1.71	-0.15	7.86	0.11	-0.14	0.06	-0.06	-0.06	0.02	0.02	0.17	-0.01	0.01	-0.07	0.06	-0.01	0.29	-				
16	Rural Population	65.75	7.23	40.86	84.19	0.04	0.13	-0.04	0.04	-0.04	-0.02	0.10	-0.01	0.02	-0.11	0.07	0.05	-0.23	0.46	-0.28	-			
17	Gender Equality (WEOI)	40.64	5.88	19.23	48.65	-0.08	0.20	-0.10	0.04	-0.01	0.02	0.01	-0.02	0.13	-0.19	0.03	0.11	0.23	-0.20	0.01	0.13	-		
18	Gender Diversity Firm Owner	0.05	0.13	0.00	0.50	0.06	0.01	-0.02	0.04	0.04	0.11	0.06	0.04	0.17	-0.34	0.15	0.15	0.02	0.03	0.09	0.07	0.11	-	
19	Gender Top Manager	0.09	0.28	0.00	1.00	0.07	-0.08	0.06	0.04	0.04	0.05	0.07	0.07	0.01	-0.03	-0.02	0.07	-0.02	0.06	0.09	-0.02	0.01	0.16	-
20	Gender Diversity Workforce	0.16	0.19	0.00	0.50	0.14	-0.20	0.12	0.07	0.08	0.10	0.07	0.16	0.04	-0.03	-0.04	0.05	-0.01	0.13	0.20	0.00	0.04	0.10	0.16

Please note that the following variables are not centred at their respective mean in this table to allow for better interpretation: firm size, gender equality, gender diversity among the owners (Blau’s Index), gender diversity in the workforce (Blau’s Index).

Table 3 presents the main results of our analysis. Model 1 is the baseline model, which exclusively contains the control variables and serves to evaluate the added explanatory value of the independent variables. Model 2 adds the three direct effects of the independent variables, namely gender diversity in the firm ownership structure (hypothesis 1a) and the overall workforce (hypothesis 3a) as well as the gender of the top manager (hypothesis 2a). Model 3 includes the three interaction effects between our independent variables and gender equality (hypotheses 1b, 2b, 3b). Table 3 reports the results of model 1 through 3. Compared to model 1, both model 2 and 3 are significant improvements in model fit. The results of the hierarchical linear models with random intercepts and random slopes (robustness test) are summarized in Table 4.

**Table 3:** Binary logistic regression models with clustered standard errors: Effect of gender diversity and the interaction effect between gender diversity and gender equality on innovation (n = 18 547).

Variables	Model 1		Model 2		Model 3	
*Control Variables*	B	SE	B	SE	B	SE
Industry (Manufacturing)	0.10*	(0.04)	0.20**	(0.04)	0.20**	(0.04)
Industry (Retail)	0.21**	(0.06)	0.20**	(0.06)	0.20**	(0.06)
Firm Size	0.00	(0.00)	0.00	(0.00)	0.00	(0.00)
Establishment Type	0.08^+^	(0.04)	0.04	(0.04)	0.05	(0.04)
Export	0.37**	(0.04)	0.30**	(0.04)	0.31**	(0.04)
R&D	1.60**	(0.04)	1.60**	(0.04)	1.60**	(0.04)
Education	0.00**	(0.00)	0.00**	(0.00)	0.00**	(0.00)
Ownership (Shareholding)	0.10	(0.12)	0.05	(0.12)	0.05	(0.12)
Ownership (Sole Proprietorship)	0.13	(0.11)	0.15	(0.12)	0.14	(0.12)
Ownership (Partnership)	0.01	(0.12)	-0.01	(0.12)	-0.00	(0.12)
Ownership (Limited Partnership)	0.34**	(0.12)	0.32**	(0.12)	0.32**	(0.12)
Income Classification	-0.08	(0.05)	-0.09^+^	(0.05)	-0.10^+^	(0.05)
Employment in Agriculture	0.01**	(0.00)	0.00*	(0.00)	0.01*	(0.00)
Foreign Direct Investment	0.12**	(0.01)	0.10**	(0.01)	0.10**	(0.01)
Rural Population	0.01**	(0.00)	0.01**	(0.00)	0.01**	(0.00)
Gender Equality	-0.03**	(0.00)	-0.04**	(0.00)	-0.03**	(0.00)
***Direct effects of Gender Diversity***						
Gender Diversity Firm Owner			0.52**	(0.14)	0.37*	(0.15)
Gender Top Manager			0.13*	(0.06)	0.14*	(0.06)
Gender Diversity Workforce			1.13**	(0.09)	1.13**	(0.09)
***Interactions***						
Gender Equality X Diversity Firm Owner					0.08**	(0.03)
Gender Equality X Gender Top Manager					-0.01	(0.01)
Gender Equality X Diversity Workforce					-0.02	(0.02)
Constant	-2.25**	(0.23)	-2.09**	(0.23)	-2.11**	(0.23)
LR Chi2			192.59	(0.00)	202.60	(0.00)
BIC (df)	22631.4	(17)	22468.2	(20)	22487.7	(23)

^+^p<0.10,

* p<0.05,

** p<0.01

**Table 4:** Robustness test. Hierarchical linear model: effect of gender diversity and the interaction effect between gender diversity and gender equality on innovation (n = 18 547).

*Fixed Effects*	Coefficient	S.E.	Coefficient	S.E.
Industry (Manufacturing)	0.06**	(0.01)	0.06**	(0.01)
Industry (Retail)	0.04**	(0.01)	0.04**	(0.01)
Firm Size	0.00*	(0.00)	0.00*	(0.00)
Establishment Type	0.01	(0.01)	0.01	(0.01)
Export	0.05**	(0.01)	0.05**	(0.01)
R&D	0.36**	(0.01)	0.36**	(0.01)
Education	0.00**	(0.00)	0.00**	(0.00)
Ownership (Shareholding)	0.02	(0.03)	0.02	(0.03)
Ownership (Sole Proprietorship)	0.02	(0.02)	0.02	(0.02)
Ownership (Partnership)	0.01	(0.03)	0.01	(0.03)
Ownership (Limited Partnership)	0.06*	(0.02)	0.06*	(0.02)
WEOI	-0.00	(0.00)	-0.00	(0.00)
Gender Diversity Ownership	0.12*	(0.05)	0.12*	(0.05)
Gender Top Manager	0.02^+^	(0.01)	0.02^+^	(0.01)
Gender Diversity Workforce	0.17**	(0.05)	0.15**	(0.04)
Gender Equality*Gender Diversity Ownership			-0.00	(0.01)
Gender Equality*Gender Top Manager			0.00	(0.00)
Gender Equality*Gender Diversity Workforce			-0.01*	(0.01)
***Random Effects***				
Variance intercept	0.01	(0.00)	0.01	(0.00)
Variance random slope Gender Diversity Ownership	0,01	(0.01)	0,02	(0,02)
Variance random slope Gender Top Manager	0.00	(0,00)	0.00	(0,00)
Variance random slope Gender Diversity Workforce	0.02^+^	(0,01)	0.02^+^	(0,01)
Log-likelihood	-11521,106		-11518,621	

^+^p<0.10,

* p<0.05,

** p<0.01

### Direct effect of gender diversity on innovation

Model 2 of the binary logistic regression analysis (see Table 3) describes the direct effect of the independent variables on innovation. The results suggest that a gender diverse ownership and workforce structure as well as a female top manager facilitate higher innovation levels. As such, the results provide strong support for hypotheses 1a, 2a and 3a. To enable a better understanding of the effect sizes, we follow the common practice of examining the marginal effects of the independent variable at one standard deviation below and above the mean [71].

Moving from one standard deviation below to one standard deviation above the mean in gender diversity among owners increases firms’ likelihood to innovate by 21.42 percent. Furthermore, we find that having a female top manager increases the likelihood of firms to innovate by 2.19 percent. Finally, moving from one standard deviation below to one standard deviation above the mean in gender diversity among a firm’s workforce gives rise to its innovation likelihood by 47.24 percent.

In terms of effect magnitude, the analyses clearly show that diversity in the workforce has the largest effect on firm innovativeness. Its effect is double that of diversity in the ownership of a company and more than 20 times as big as the effect of the gender of the top manager.

### Moderating effect of gender equality

Model 3 assesses the interaction effect between gender diversity and gender equality, shedding light on the three hypothesized moderation effects (Hypotheses 1b, 2b, 3b). Contrary to our expectations, the results of the binary logistic regression and the hierarchical linear regression (see Table 4) for the proposed moderation effect of gender equality are mostly non-significant as well as inconsistent. As such, our results thus do not support the hypotheses that gender equality moderates the relationship between gender diversity and innovation.

More specifically, hypothesis 1b is only partially supported. Whereas the binary logistic regression (Table 3) suggests gender equality to moderate the relationship between gender diversity among a firm’s ownership structure and its likelihood to innovate, the hierarchical linear model (Table 4) yields in non-significant results. As such, we cannot infer that our results support hypothesis 1b.

Based on the results of both model specifications, hypothesis 2b is not supported. There is insufficient evidence at a 5 percent significance level to reject the claim that gender equality has no effect on the relationship between the gender of a firm’s top manager and its likelihood to innovate.

Similar to hypothesis 1b, the findings of hypothesis 3b are inconsistent. Whereas our main model (binary logistic regression, Table 3) suggests that gender equality has a non-significant impact on the relationship between gender diversity among the workforce and innovation, the hierarchical linear model (Table 4) proposes a significant negative moderation effect. Based on the inconsistency across the two model specifications, there is insufficient evidence to support the hypothesis that gender equality moderates the relationship between gender diversity and innovation.

### Endogeneity

The Enterprise Survey is restricted to cross-sectional observations. Therefore, unobserved heterogeneity and self-selection are a concern for our study [72]. We were able to address these concerns for two of our three independent variables. First, we performed instrumental variable probit regressions, in which we instrumented diversity among the workforce in our firms. We instrumented with the industry-specific average gender diversity in the workforce in the Netherlands. This approach of using industry averages from another country as an instrument is often used in studies on firm-level innovation as it provides highly exogenous instruments [73]. Following the procedure described by Bascle (2008), we checked whether the instrument is sufficiently strong. We did so by comparing the first-stage F statistic to Stock and Yogo’s (2005) critical values. The F-statistics for model 4 and 5 (see Table 5) both pass the highest critical value (5% maximal IV relative bias) and we therefore conclude that our instruments are sufficiently strong. Importantly, the main effect of gender diversity in the workforce remains positive and highly significant indicating that our findings in this regard are not an artifact of endogeneity. Moreover, model 5 reveals a positive and statistically significant interaction effect between gender diversity in the workforce and gender equality. This effect, which is in-line with hypothesis 3b was neither present in our main analyses nor in the hierarchical linear models used as a robustness test. We take this finding as further evidence that the interaction between gender diversity and gender equality is rather sensitive to model specifications, but that the positive effect of gender diversity on innovativeness is not.

**Table 5:** Instrumental variable regressions for gender diversity in the workforce (n = 18 547).

Variables	Model 4		Model 5	
*Control Variables*	B	SE	B	SE
Industry (Manufacturing)	0.203**	0.037	0.198**	0.037
Industry (Retail)	0.116**	0.035	0.090*	0.038
Firm Size	0.000	0.000	0.000	0.000
Establishment Type	0.011	0.028	0.000	0.028
Export	0.124**	0.032	0.104**	0.033
R&D	0.96**	0.025	0.961**	0.025
Education	0.02**	0.000	0.002**	0.000
Ownership (Shareholding)	-0.011	0.078	-0.019	0.079
Ownership (Sole Proprietorship)	0.042	0.073	0.025	0.074
Ownership (Partnership)	-0.034	0.074	-0.043	0.075
Ownership (Limited Partnership)	0.173*	0.075	0.150*	0.076
Income Classification	-0.09**	0.034	-0.030	0.046
Employment in Agriculture	0.000	0.002	0.002	0.002
Foreign Direct Investment	0.047**	0.009	0.039**	0.009
Rural Population	0.06**	0.002	0.004*	0.002
Gender Equality	-0.03**	0.002	-0.026**	0.002
***Direct effects of Gender Diversity***				
Gender Diversity Firm Owner	0.217*	0.086	0.187*	0.088
Gender Top Manager	0.010	0.043	0.026	0.045
Gender Diversity Workforce^a^	1.761**	0.348	1.797**	0.350
***Interactions***				
Gender Equality X Diversity Workforce^a^			0.135+	0.0690
Model significance	0.000		0.000	
First-stage F statistic	179.85		43.61	

^+^p<0.10,

* p<0.05,

** p<0.01

^a^: instrumented variable

Second, we ran a propensity score matching model to deal with endogeneity for the gender of the top manager. This technique extracts ‘treatment effects’ from observational data [74]. In our case, the ‘treatment’ is a company having a female top manager. We matched companies receiving the ‘treatment’ matched to four other companies that are as similar as possible on all observed covariates used in our analyses, but that did not receive the treatment (i.e., had a male top manager) [75]. A comparison of these matched cases reveals a remaining sizeable and statistically significant effect (treatment effect = 0.045, p<0.01) of having a female top-manager of innovativeness. Again, this insight provides evidence that our findings in this regard are not an artifact of endogeneity.

## Discussion

Despite the varying and generally low levels of gender equality in developing countries, we find that gender diversity among a firm’s owners and workforce, as well as a female top manager, have a significant positive effect on innovation. Our results furthermore propose that innovation benefits from gender diversity independent of gender equality. As such, our findings broaden and refine existing knowledge on gender diversity and innovation, with both theoretical as well as practical implications.

First, we suggest a more universal applicability of gender diversity as a means to increase innovation beyond the previously set boundaries of the United States and European countries, where most diversity research has been conducted to date [29]. The results of our study put forward that gender diversity at all organizational levels as well as having a female top manager have a direct positive effect on a firm’s likelihood to innovate in the developing countries participating in our study. We thus identify an important firm-level mechanism for increasing innovation in developing countries in Africa, the Middle East and South Asia as well as enhance the scholarly understanding of how ‘understudied phenomena influence existing theories’ [19]. Given the binary nature of our dependent variable (product innovation), future research should assess whether the effects found in this study also hold true for different types of innovation (e.g. process innovation) as well as for different forms of innovation (radical vs incremental innovation). Furthermore, a continuous measurement of innovation may provide more fine-grained insights.

Second, our study puts forward that the positive effect of gender diversity among a firm’s owner and workforce as well as of having a female top manager is independent of a countries level of gender equality (contradicting hypotheses 1b, 2b, 3b). Based on the insights of this research, future studies in developing countries could assess whether the influence of female owners, employees and top managers on innovation ‘may be moderated by a firm’s attitudes toward’ women [76] rather than by a country’s level of gender equality. Support for this line of thought is provided by recent studies conducted in developed countries. For example, Reinwald and colleagues [77] suggest that a strong diversity climate can positively impact firm performance. Furthermore, also following a contingency approach, Dwyer and colleagues find that organizational culture moderates the effectiveness of gender diversity for firm performance [78]. Moreover, Wang and Kelin [79] propose that the success of women at different hierarchical levels is interdependent. It would be interesting for follow-up studies to both extend previous insights to developing countries as well as to further refine them.

Our results also have practical implications. We provide empirical evidence of the importance gender diversity has for innovation and thus economic growth in developing countries. Thereby our research channels a powerful message that supports not only the societal and ethical rationale for women’s inclusion in the workplace, but also showcases the therewith associated business advantages for firms in developing countries. Our results encourage firms to actively increase their percentage of women. Effective implementation of such policies can include human resource management, such as targeted hiring, as well as equal access to training or career development for women [20]. It is important that the heterogeneity among women on the one side as well as the country-specific constraints they are faced with on the other side are taken into consideration when designing respective programs [80]. Our results also have implications for policy makers. As innovation enables economic development [9] and as gender diversity benefits innovation, policy makers are encouraged to support firms in their efforts to increase gender diversity. Efforts can include financial incentives as well as setting quota for female participation in the workforce, among firm owners as well as appointing women as top managers [81]. As the magnitude of the direct positive effect of gender diversity among the workforce on innovation is highest, we propose a special focus on increasing women’s participation in the overall workforce.

## Conclusion

To conclude, the results of our study suggest that gender diversity in developing countries in Africa, the Middle East and South Asia is not only important from an ethical perspective, but also from an economic point of view. Gender diversity can increase firms’ likelihood to innovate, despite their comparably low levels of gender equality. The results of our study thus suggest that a phenomenon, which has to date been primarily studied and benefited from in developed countries, is also applicable in the context of developing countries. The implications of our findings are relevant both for firms as well as for policy makers. Firms are encouraged to aim at establishing gender balance at all levels of the firm to increase the likelihood for innovation. Ideally, this would additionally be motivated and, if required, enforced by corresponding country legislation prompting firms to create equal employment opportunities for men and women.

## Supporting information

S1 Appendix Summary of WEOI indicators. (DOCX)
