# Supplementary material for: Is this a man’s world? The effect of gender diversity and gender equality on firm innovativeness
Source: PLoS One. 2019 Sep 18;14(9):e0222443. doi: 10.1371/journal.pone.0222443 (PMC6750582; doi:10.1371/journal.pone.0222443)
Supplement: S1 Appendix — (DOCX) [file pone.0222443.s001.docx]

**Appendix 1. Summary of WEOI indicators.**

| **Category** | Description of indicators and underlying variables |
| --- | --- |
| **Labour Policy** | Equal pay for equal Work: ILO Equal Remuneration Convention (No 100); country progress in aligning national policy with the terms of the Convention; 8 latent variables |
|  | Non-discrimination: ILO Discrimination (Employment and Occupation) Convention (No 111); country progress in aligning national policy with the terms of the Convention; 9 latent variables |
|  | Maternity and paternity leave provision: a composite indicator that assesses the length of maternity and paternity leave, and maternity benefits coverage stipulated per law; score 1-9 |
|  | Legal restrictions on job types for women; a composite indicator that incorporates 8 restrictions imposed on women; score 1-8 |
|  | Difference between the statutory (pensionable) retirement age between men and women |
| **Labour Practice** | Equal pay for equal Work: ILO Equal Remuneration Convention (No 100); country progress in enforcing the provisions of the Convention; 6 latent variables |
|  | Non-discrimination: ILO Discrimination (Employment and Occupation) Convention (No 111); country progress in enforcing the provisions of the Convention; 5 latent variables |
|  | Degree of *de facto* discrimination against women in the workplace (equal opportunities) |
|  | Availability, affordability and quality of childcare services, as well as the role of the extended family in providing childcare |
| **Access to finance** | Building credit histories; a composite measure of the ability to build a credit history; score 1-6 |
|  | Women's access to finance programs; composite indicator capturing 3 latent variables; score 1-5 |
|  | Delivering financial services; composite indicator based on 3 latent variables; score 1-5 |
|  | Private-sector credit as a percentage of Gross Domestic Product; capturing levels of credit availability and sustainability; score 1-3 |
| **Education and training** | School life expectancy (primary and secondary) for women; average years of schooling |
|  | School life expectancy (tertiary) for women; average years of schooling |
|  | Mean years of schooling; average years of schooling |
|  | Adult literacy rate of women; percentage |
|  | Existence of government or non-government programmes offering small and medium-sized enterprise (SME) support/development training; score 1-5 |
| **Women’s legal and social status** | Law addressing violence against women; 4 latent variables (domestic violence, sexual assault/ violence and rape, sexual harassment); score 0-4 |
|  | Citizenship rights; 3 latent variables (freedom of movement, dress code in public, access to passport); score 0-3 |
|  | Property ownership rights; equal ownership rights for men and women over for property both by law and in practice; score 0-5 |
|  | Adolescent fertility rate; age-specific fertility rate per 1,000 women, 15-19 years of age |
|  | Prevalence of contraceptive use, modern methods |
|  | Country ratification of the Convention on the Elimination of All Forms of Discrimination against Women (CEDAW); score 0-4 |
|  | Political participation (percentage of women in ministerial positions, and in parliament) |
| **Regulatory quality** | Composite measure for starting a business; 4 latent variables (procedures, duration, cost and paid-in minimum capital for starting a business) |
|  | Infrastructure risk; composite measure to assess whether infrastructure meats business needs |
|  | Access to technology and energy; percentage of population with access to mobile phones, internet, water, sanitation, electricity |
